# Alkyl Chain Growth on a Transition Metal Center: How Does Iron Compare to Ruthenium and Osmium?

**DOI:** 10.3390/ijms161023369

**Published:** 2015-09-28

**Authors:** Mala A. Sainna, Sam P. de Visser

**Affiliations:** Manchester Institute of Biotechnology and School of Chemical Engineering and Analytical Science, the University of Manchester, 131 Princess Street, Manchester M1 7DN, UK; E-Mail: alhaji.mala@manchester.ac.uk

**Keywords:** density functional theory, hydrogen atom abstraction, catalysis, carbon monoxide, alkanes, methyl transfer, thermodynamics

## Abstract

Industrial Fischer-Tropsch processes involve the synthesis of hydrocarbons usually on metal surface catalysts. On the other hand, very few homogeneous catalysts are known to perform a Fischer-Tropsch style of reaction. In recent work, we established the catalytic properties of a diruthenium-platinum carbene complex, [(CpRu)_2_(μ^2^-H)(μ^2^-NHCH_3_)(μ^3^-C)PtCH_3_(P(CH_3_)_3_)_2_](CO)_*n*_^+^ with *n* = 0, 2 and Cp = η^5^-C_5_(CH_3_)_5_, and showed it to react efficiently by initial hydrogen atom transfer followed by methyl transfer to form an alkyl chain on the Ru-center. In particular, the catalytic efficiency was shown to increase after the addition of two CO molecules. As such, this system could be viewed as a potential homogeneous Fischer-Tropsch catalyst. Herein, we have engineered the catalytic center of the catalyst and investigated the reactivity of trimetal carbene complexes of the same type using iron, ruthenium and osmium at the central metal scaffold. The work shows that the reactivity should increase from diosmium to diruthenium to diiron; however, a non-linear trend is observed due to multiple factors contributing to the individual barrier heights. We identified all individual components of these reaction steps in detail and established the difference in reactivity of the various complexes.

## 1. Introduction

With dwindling natural supplies of oil and gas and the threat that these resources will run out in the next Century, attempts have been made to find ways of generating oil and gas synthetically. Advances of these alternatives were already made before the Second World War when Fischer and Tropsch discovered a chemical process to combine CO and H_2_ gas under high temperature and pressure on metal surfaces to form linear alkanes [1]. This so-called Fischer-Tropsch process was an alternative means to generate oil and gas and well-used and applied by Germany in that period of time, due to its lack of access to natural oil. After the war ended, and supply of natural oil and gas became available again, the interest in the Fischer-Tropsch processes lessened as a result of the considerable price difference between natural oil and synthetically created oil. In recent years, however, a revival of the Fischer-Tropsch process has begun mainly as a consequence of reduced stocks of natural oil and gas. The main problem that prevents the Fischer-Tropsch processes from extensive use in Industry is its difficulties in generating alkanes efficiently at low cost. Thus, the chemical system uses high temperatures and pressures and often takes place on an expensive heterogeneous catalyst [2,3,4,5,6,7,8]. As such, the process cannot compete efficiently with natural oil and gas extraction.

**Scheme 1:**
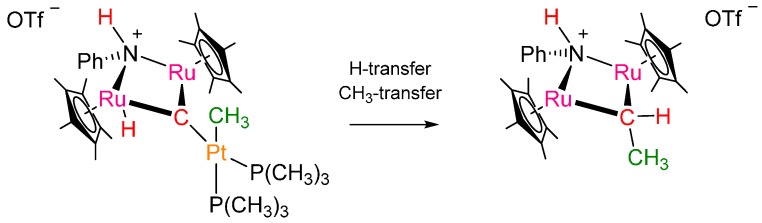
Basic features of the trimetal carbene Fischer-Tropsch complex studied here.

In order to make advances with the Fischer-Tropsch process, attempts have been made to create homogeneous catalysts in solution [9,10]. Unfortunately, currently there are only a handful or so homogeneous Fischer-Tropsch catalysts available and none are competitive with either heterogeneous catalysts or natural oil production. Some years ago, Matsuzaka and co-workers developed and designed a unique trimetal carbene catalyst that was found to be able to generate linear alkanes in a toluene solution [11]. The basic features of the Matsuzaka catalyst are given in Scheme 1. It contains a central carbene, linked to two Ru atoms and one Pt atom. In this very rigid structure, a hydrogen atom is transferred from Ru to the carbene followed by a methyl transfer from Pt to form the ethylidene moiety and splits off the Pt(P(CH_3_)_3_)_2_ group. Recently, our group performed a series of density functional theory calculations on this reaction mechanism, with and without additional CO [12]. It was found that CO has a dramatic effect on the reaction rates and overall mechanism. The calculations showed that although an initial hydrogen atom transfer is low in energy, actually the subsequent barrier is very high and, therefore, the reverse order of initial methyl transfer followed by hydrogen atom transfer is energetically favorable. Nevertheless, the reaction uses expensive catalysts such as Ru and Pt and will be difficult to commercialize. To further gain insight into this unique trimetal carbene system, we decided to investigate two alternative catalysts, whereby the two Ru atoms are replaced by either Os or Fe. This enables us to understand the effect of the transition metal on the catalytic activity on a trimetal carbene center.

## 2. Results and Discussion

In this work we investigated the catalytic properties of the complex shown in Scheme 1 and two modified systems where the two ruthenium atoms were replaced by either iron or osmium. To reduce the computational cost of the calculations, in our models the phenyl substituent was abbreviated to methyl and the η^5^-C_5_(CH_3_)_5_ groups with η^5^-C_5_H_5_ instead. In addition, we studied one system with two CO molecules bound to the metal centers and another one without CO, because in previous work major differences in geometry and reactivity were obtained [12]. The calculations focused on the pathways leading to ethylidene formation from the reactant complex, which involves a hydrogen atom and methyl transfer to the central carbene atom. We particularly focus on the order of the hydrogen and methyl transfer processes and the effect of CO on the mechanism and energetics.

**Figure 1:**
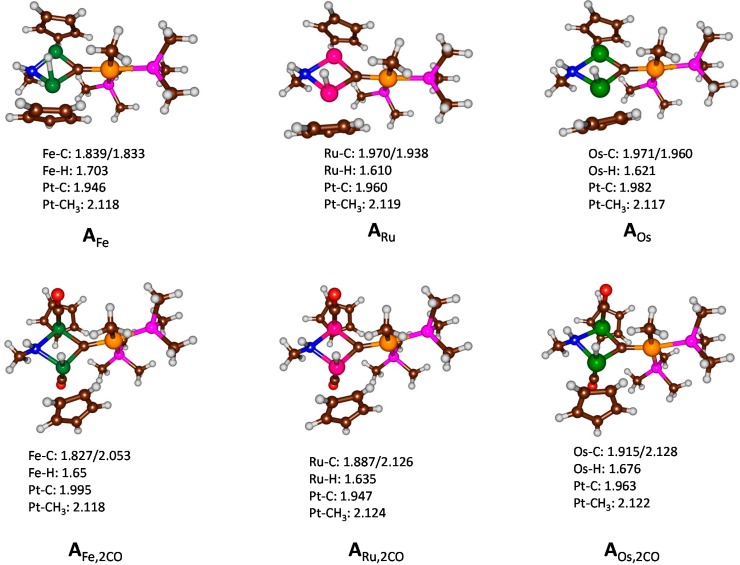
Optimized geometries of reactant complexes without CO (**top row**) and with 2 CO molecules bound (**bottom row**) as obtained by density functional theory (DFT). Geometries optimized with UB3LYP/BS1 and selected bond lengths are given in angstroms.

In the first set of calculations, we investigated the reactant structure, namely [(CpM)_2_(μ^2^-H)(μ^2^-NHCH_3_)(μ^3^-C)PtCH_3_(P(CH_3_)_3_)_2_](CO)_*n*_^+^ or **A**_M_ with M = Fe, Ru or Os and *n* = 0 or 2. Figure 1 shows the optimized geometries of **A**_Fe_, **A**_Ru_, **A**_Os_, **A**_Fe,2CO_, **A**_Ru,2CO_, and **A**_Os,2CO_. The top row of structures in Figure 1 gives those without CO bound, whereas the bottom row gives the optimized geometries with two complexed CO molecules. The overall shape and structure does not appear to have undergone major changes upon CO binding, but a few selected changes can be observed. Specifically, one of the metal-carbene distances is well over 2 Å in the complexes with CO bound, whereas the two carbene-metal (Fe/Ru/Os) distances are virtually identical in the complexes lacking CO molecules. As a consequence, the hydrogen atom bound to these metals is located in between the two metal ions in the structures without CO bound. In particular, in complex **A**_Fe_ the hydrogen atom is almost on the line in between the two iron atoms, although it is closer to one atom in the Ru and Os cases. Attempts to optimize a bridging hydrogen atom failed and the calculation converged back to the structures shown in Figure 1. Therefore, the system with a bridging hydrogen atom is likely to be an excited state.

Structurally all geometries look very similar especially regarding the ligand structure, and, for instance, all Pt–CH_3_ distances are within 0.007 Å, which implicates a minor effect on the Pt binding properties upon changing the transition metals. Most optimized geometries give bond lengths as expected from calculations on analogous complexes [13,14,15]. In the case of **A**_Fe,2CO_ a full geometry optimization without constraints led to hydrogen atom transfer from Fe to the carbene center, which implies a low-barrier process for hydrogen transfer. In the end we optimized structure **A**_Fe,2CO_ with a fixed Fe–H distance of 1.65 Å, which converged to the structure shown in Figure 1.

**Figure 2:**
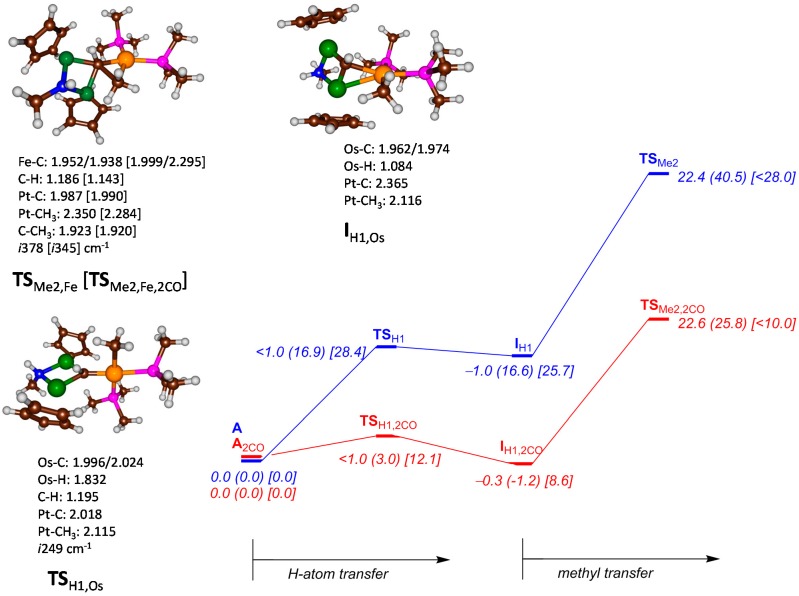
Potential energy landscape for ethylidene formation from complex **A** with initial hydrogen abstraction followed by methyl transfer. All energies are obtained with UB3LYP/BS2 and contain zero-point and solvent corrections in kcal·mol^−1^. Optimized geometries of key structures are given with bond lengths in angstroms. The blue surface represents the bare system without CO, whereas the red surface contains the CO bound complexes. Energy data is given for Fe (Ru) [Os] complexes.

Subsequently, we investigated the methyl and hydrogen atom transfer reactions of all complexes to form ethylidene products. We investigated two possible mechanisms, namely (*i*) first hydrogen atom transfer from **A** followed by methyl transfer to form CHCH_3_ products and (*ii*) first methyl transfer followed by hydrogen atom transfer to give CHCH_3_ products. As the regioselectivity of aliphatic hydrogen atom abstraction *versus* electrophilic reactivities is sometimes affected by the ligand architecture of the metal ion [16,17,18,19], we considered complexes with and without CO bound. Figure 2 displays the pathway for initial hydrogen atom transfer from M (M = Fe, Ru, Os) to the carbene atom and is followed by methyl transfer from Pt. Pathway (*i*) is stepwise with an initial hydrogen atom abstraction barrier **TS**_H1_ to form the H-transfer intermediate **I**_H1_ and followed by methyl relay via a transition state **TS**_Me2_ to form products **P**. Pathway (*ii*) takes place with an initial methyl transfer from Pt in **A** via transition state **TS**_Me1_ to form the methyl-transfer intermediate **I**_Me1_ and in the next step a proton is transferred from the metal via transition state **TS**_H2_ to form products. Figure 2 displays relative energies obtained for pathway (*i*) for all three metal complexes. As discussed above, the iron system has negligible or small hydrogen atom transfer barriers either with or without bound CO and efficiently leads to H-transfer intermediates **I**_H1_. However, a significant barrier exists of 22.4 (22.6) kcal·mol^−1^ with (without) bound CO, which makes this step rate determining. Optimized geometries of these two transition states (**TS**_Fe,Me2_) look very similar and are characterized by an imaginary frequency of *i*378 (*i*345) cm^−1^ for the C–C stretch (bond formation) vibration for **TS**_Fe,Me2_ (**TS**_Fe,Me2,2CO_). The iron landscape looks very similar to that found for ruthenium, but is shifted downwards, and, hence the **TS**_Me2_ barrier, which was inaccessible at room temperature for ruthenium is now possible for iron. A change from ruthenium to osmium, by contrast, raises all energies and makes it a lesser suitable catalyst for hydrogen and methyl transfer processes. In particular, the hydrogen atom transfer barriers are high in absence of CO (28.4 kcal·mol^−1^), which drop dramatically when CO molecules are added to the complex (to 12.1 kcal·mol^−1^).

**Figure 3:**
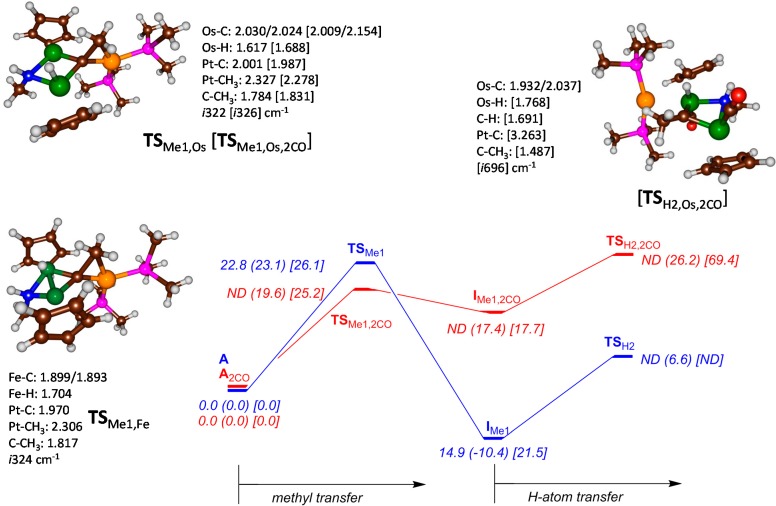
Potential energy landscape for ethylidene formation from complex **A** with initial methyl transfer followed by hydrogen transfer. All energies are obtained with UB3LYP/BS2 and contain zero-point and solvent corrections in kcal·mol^−1^. Optimized geometries of key structures are given with bond lengths in angstroms. ND stands for not determined. The blue surface represents the bare system without CO, whereas the red surface contains the CO bound complexes. Energy data is given for Fe (Ru) [Os] complexes.

To find out whether the alternative order of methyl followed by hydrogen transfer pathway would be feasible, we also investigated initial methyl transfer followed by hydrogen abstraction and the results are given in Figure 3. The reaction is stepwise with an initial methyl transfer barrier **TS**_Me1_ to form the methyl-transfer intermediate **I**_Me1_. This step is followed by hydrogen atom transfer via a transition state **TS**_H2_ to form products **P**. As mentioned above, we had difficulties in optimizing structure **A**_Fe_ and **A**_Fe,2CO_ due to the low-energy H-transfer barrier. Therefore, we manually generated **I**_Fe,Me1_, and after geometry optimization performed a backwards geometry scan in the direction of reactants. This enabled us to characterize **TS**_Fe,Me1_, although the structure with 2 CO molecules still failed to converge and fell back to **I**_H1,Fe,2CO_. These studies implicate that with two iron atoms in the catalytic center, the only possible reaction pathway will be the ones displayed in Figure 2 with an initial hydrogen atom abstraction followed by methyl transfer to form ethylidene products.

The barrier heights obtained for **TS**_Me1_ and **TS**_Me1,2CO_ for the series Fe, Ru and Os give a slightly increase in barrier height upon choosing a larger metal ion. We predict, therefore, a value of **TS**_Fe,Me1,2CO_ slightly lower than 19.6 kcal·mol^−1^, which is significantly higher in energy than the almost barrierless hydrogen transfer. As such the reaction starting with methyl transfer will not be able to compete with initial hydrogen transfer at least for the case of iron. In the case of osmium, a similar pattern is observed, whereby the reaction starting with an initial hydrogen atom transfer has a low barrier (12.1 kcal·mol^−1^ with 2 CO molecules added). Although the reaction starting with methyl transfer is 13.4 kcal·mol^−1^ higher in energy, actually the subsequent barrier is so high (69.4 kcal·mol^−1^) that this pathway can be considered unlikely. Optimized geometries are similar to those observed previously on the ruthenium complex and the transition states give an imaginary frequency for the correct mode [12].

In order to validate the computational energetics and test the reproducibility of the computational methods, we did a series of test calculations with alternative density functional methods, *i.e.*, B3LYP-D3 and M06-L, on the optimized geometries. Table 1 gives relative energies (ΔE + ZPE) obtained this way. As can be seen only very minor differences in relative energies are obtained and all values are within a few kcal·mol^−1^. Clearly, the chemical catalyst described here shows little sensitivity to the density functional method, but also the chemical system appears very stable despite environmental and dispersion perturbations. Note here that free energy, entropy, solvent and dispersion corrections had very little effect on the relative energies.

**Table 1:** Relative energies obtained with basis set BS2 through single point calculations on B3LYP/BS1 optimized geometries. All energies are in kcal·mol^−1^ and contain ZPE corrections.

Structure	B3LYP	B3LYP-D3	M06-L
**A**_Fe_	0.00	0.00	0.00
**TS**_Me1,Fe_	24.31	24.69	23.89
**I**_Me1,Fe_	17.92	18.44	17.86
**I**_H1,Fe_	5.21	5.91	9.07
**TS**_Me2,Fe_	24.88	25.87	34.57
**A**_Os_	0.00	0.00	0.00
**TS**_Me1,Os_	26.63	25.63	23.88
**I**_Me1,Os_	22.80	20.97	19.30
**TS**_H1,Os_	28.33	30.05	25.16
**I**_H1,Os_	26.89	26.36	20.01
**A**_Os,2CO_	0.00	0.00	0.00
**TS**_Me1,Os,2CO_	25.79	24.70	23.30
**I**_Me1,Os,2CO_	18.92	18.08	15.70
**TS**_H1,Os,2CO_	11.44	11.69	10.70
**I**_H1,Os,2CO_	8.35	9.47	7.06

The calculations presented in this work give dramatic differences in catalytic activity even though only two atoms have been replaced by atoms in the same column of the periodic table. Thus, in the case of the ruthenium complex it was shown that an initial hydrogen transfer was energetically favorable over initial methyl transfer. However, the subsequent step after hydrogen transfer was found to be very high in energy and unable to lead to products at room temperature so that the only feasible pathway leading to products was the one starting with initial methyl transfer [12]. By contrast, the iron and osmium pathways have low barriers for the full mechanism starting with hydrogen abstraction and will not proceed with initial methyl transfer instead.

To explain the features of the competitive hydrogen atom *versus* methyl transfer processes, we set up a valence bond (VB) curve crossing diagram. These diagrams have been used extensively in the past to rationalize trends in, e.g., hydrogen atom abstractions from iron(IV)-oxo complexes, double bond epoxidation by iron(IV)-oxo complexes and sulfoxidation reactions by iron(IV)-oxo complexes [19,20,21,22,23,24,25]. The VB diagram starts at the center with a VB structure of the reactant complex, which has key bonds highlighted with a line shared by two dots that represent two electrons in a chemical bond. There are bonds between the carbene atom and the two metal ions (Fe, Ru or Os) and a third to the Pt atom. One of the metal ions holds the hydrogen atom and the methyl group is linked to Pt. An initial hydrogen atom transfer (pathway from the center to the left) leads to the **I**_H1_ intermediates, where the carbene picks up the hydrogen atom from Fe/Ru/Os and the methyl group is still attached to Pt. A VB diagram assumes that the wave function of the reactants, which is Ψ_R_ for **A**_M_/**A**_M,2CO_, is connected to an excited state in the products, *i.e.*, **I**_H1_ in our case. At the same time, the product wave function leads to an excited state in the reactant configuration. In our case, two possible reaction pathways were investigated, hence there is an excited state representing the hydrogen transfer mechanism (with wave function Ψ_H_*) and one for methyl transfer (with wave function Ψ_Me_*). The excitation energy G_HT_ for the excitation Ψ_R_→Ψ_H_* is proportional to the hydrogen atom abstraction barrier (ΔE_H_^‡^), whereas the excitation energy (G_MT_) for the excitation Ψ_R_→Ψ_H_* would be proportional to the methyl transfer barrier (ΔE_Me_^‡^). A careful analysis of the bond breaking and bond formation processes will give hints on the origins of the barrier heights as well as the trends observed.

Let us first compare the VB structures of **A**_M_ and **I**_H1_. For simplicity we ignore the bound CO molecules as they do not contribute to the electronic factors that determine the barrier heights but give stereochemical interactions that give an additional effect. Thus, the central carbon atom is in a carbene configuration with sp^2^ hybridization. Upon hydrogen atom transfer the carbon rehybridizes to sp^3^ configuration and forms a bond with the incoming hydrogen atom. This rehybridization energy will cost the system an amount of energy E_ex,C_, whereas the energy to break the M–H bond will be equal to its bond dissociation energy (BDE_MH_). Of course, energy is gained through the formation of a C–H bond with bond dissociation energy BDE_CH_. Finally, an electron is transferred from the metal (Fe/Ru/Os) that initially held to hydrogen atom to the platinum atom, which is reduced with a value ET_M→Pt_. The total excitation energy G_HT_ can then be described through Equation (1) in terms of BDE_CH_, BDE_MH_, ET and E_ex,C_.

(1) $$G_{\text{HT}}={\text{BDE}}_{\text{MH}}-{\text{BDE}}_{\text{CH}}+E_{\text{ex}.C}+{\text{ET}}_{M\to \text{Pt}}$$

(2) $$G_{\text{MT}}={\text{BDE}}_{{\text{PtCH}}_{3}}-{\text{BDE}}_{{\text{CCH}}_{3}}+E_{\text{ex}.C}$$

**Figure 4:**
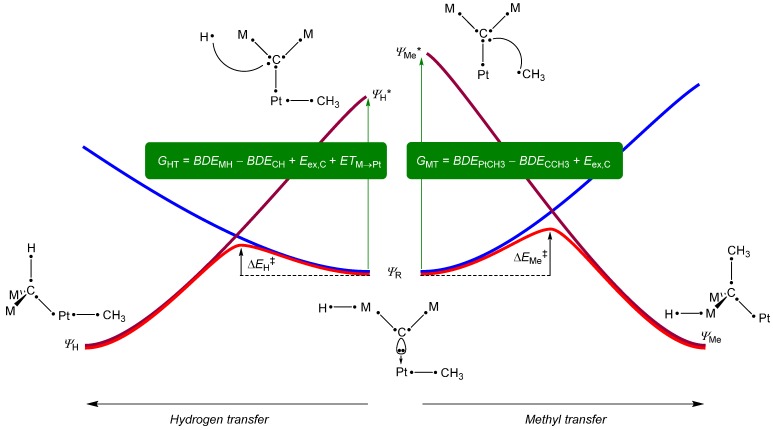
Valence bond curve crossing diagram that accounts for bond-breaking, bond-formation and electron-transfer processes in the transition states. Dots represents electrons and a line (curved or straight) represents a chemical bond.

An analysis of the reactant and methyl transfer structures (**A**_M_ and **I**_Me1_) on the right-hand-side of Figure 4 will give information on the nature of the methyl transfer processes and its barriers. As can be seen, also in this process the carbene atom is rehybridized from sp^2^ to sp^3^ and hence the barrier will also be proportional to E_ex,C_. In addition, the bond between Pt and methyl breaks, which has a bond dissociation energy ${\text{BDE}}_{{\text{PtCH}}_{3}}$ and a bond is formed between carbene and methyl with bond dissociation energy ${\text{BDE}}_{{\text{CCH}}_{3}}$. The overall excitation energy G_MT_ for the process from reactants to the methyl transfer wave function can be described by Equation (2). Previously, we calculated free energy values of 45.5 and 34.5 kcal·mol^−1^ for ${\text{BDE}}_{{\text{PtCH}}_{3}}$ for **A**_Ru_ and **A**_Ru,2CO_ [12] We do not expect these values to dramatically change when the Ru atoms are replaced by either Fe or Os as this bond is at a relatively large distance of the two metal ions. In addition, free energies for BDE_CH_ = 107.4 kcal·mol^−1^ and ${\text{BDE}}_{{\text{CCH}}_{3}}$ = 72.8 kcal·mol^−1^ were calculated for **I**_Ru,H1_ and **I**_Ru,Me1_. Finally, the Ru–H bond strength was calculated to be 66.0 and 8.0 kcal·mol^−1^ for the systems without and with two CO molecules, respectively. Clearly, the binding of CO weakens the metal-hydrogen bond strength and should lower the barriers of hydrogen atom transfer, as indeed is seen in Figure 3.

The VB analysis of Figure 4 and Equations (1) and (2) shows that the hydrogen transfer transition state is dependent on variables that are metal-dependent and metal-independent. Obviously, the metal-independent factors will not change upon replacing the two Ru atoms with other elements. For instance, we do not expect major changes in ${\text{BDE}}_{{\text{CCH}}_{3}}$ and ${\text{BDE}}_{{\text{PtCH}}_{3}}$ as the Ru ions (or their replacements) do not change during those bond formation processes. This is further supported by the very small changes in Pt–CH_3_ bond lengths of the six reactant complexes, see Figure 1. Indeed, the barrier heights for methyl transfer for the three metal complexes are very similar and the three **TS**_Me1_ barriers are found within a small window of 3.1 kcal·mol^−1^. On the other hand, the **TS**_H1_ barriers depend on the strength of the metal-hydrogen bond (BDE_MH_) as well as the electron transfer from metal to platinum (ET_M→Pt_). We expect the Fe–H bond strength to be considerably weaker than the Ru–H or Os–H bond and, therefore, predict the iron based barriers to be the lowest in energy. This prediction is in good agreement with the obtained potential energy landscapes in Figure 3 and Figure 4.

## 3. Experimental Section

The calculations reported in this work use density functional theory (DFT) methods as implemented in the Gaussian-09 program package [26]. We utilized the hybrid density functional method UB3LYP [27,28] for all studies as it was shown to reproduce experimental rate constants and free energies of activation of homogeneous catalysts well [29,30,31,32,33,34,35]. All geometries were initially optimized in Gaussian with the LANL2DZ basis set on the metals and 6-31G on the rest of the atoms: Basis set BS1 [36]. This combination of method and basis set was used for geometry optimizations, frequencies, geometry scans and intrinsic reaction coordinate studies. All local minima had real frequencies only and the transition states were characterized with a single imaginary frequency for the correct mode. Finally, single point calculations were performed with a triple-zeta quality basis set on all atoms: LACV3P+ on the metals and 6-311+G* on first and second row elements, Basis set BS2. Test calculations were done for selected systems, whereby geometries were optimized with basis set BS2, but only minor differences in the structures and minimal (<0.1 kcal·mol^−1^) in the relative energies along the potential energy landscape was observed [37,38]. The effect of solvent was investigated with single point calculations on the optimized structures using the polarized continuum model with a dielectric constant mimicking toluene. To test the reproducibility of the B3LYP results we did a series of single point calculations on the optimized geometries using B3LYP-D3/BS2 and M06-L/BS2 [39,40], which gave the same trends and conclusions.

## 4. Conclusions

A series of computational studies are presented on a potential homogeneous Fischer-Tropsch catalyst with a trimetal carbene active center. We tested a Fe_2_CPt, Ru_2_CPt and Os_2_CPt trimetal carbene catalyst for production of ethylidene on the carbene center. The calculations show that all three catalysts should efficiently form ethylidene products, but most probably the iron based system will be the most active. The work was focused on several potential mechanisms that start with either initial hydrogen atom transfer or methyl transfer. However, the methyl transfer pathways were found to be considerably higher in energy. Furthermore, we also investigated the addition of two CO molecules to the complex. Although these two CO molecules appear to be innocent, they actually contribute to the eventual reaction mechanism by stereochemical interactions. The reaction mechanism is explained with thermodynamic and valence bond analysis. Overall, the work gives detailed insight into homogeneous Fischer-Tropsch reactivities on metal centers.
